# Supplementary material for: AffyMAPSDetector: a software tool to characterize Affymetrix GeneChip™ expression arrays with respect to SNPs
Source: BMC Bioinformatics. 2007 Jul 30;8:276. doi: 10.1186/1471-2105-8-276 (PMC1959249; doi:10.1186/1471-2105-8-276)
Supplement: Additional file 13 — AffyMAPSDetector v1 source code. AffyMAPSDetector source code. [file 1471-2105-8-276-S13.zip › AffyMAPSDetector_v1_src/doc/affychip/snpdetector/Constant.html]

Constant


|  |  |  |  |
| --- | --- | --- | --- |
| **Overview** \| **Package** \| **Class** \| **Use** \| **Tree** \| **Deprecated** \| **Index** \| **Help** | | |  |
| **PREV CLASS**   NEXT CLASS | **FRAMES**    **NO FRAMES**     **All Classes** |  |  |
| SUMMARY: NESTED \| FIELD \| CONSTR \| METHOD | DETAIL: FIELD \| CONSTR \| METHOD |  |  |


---


## affychip.snpdetector Class Constant

```
java.lang.Object
  affychip.snpdetector.Constant
```

---

public class **Constant** extends java.lang.Object

Title: Probe SNP Detector

Description: Affymetrix Gene-Probe SNP Detection

Copyright: Sunita Kumari -- Copyright (c) 2004

Company: George Mason University

**Version:**
:   1.1

**Author:**
:   Sunita Kumari

---

|  |  |
| --- | --- |
| **Field Summary** | |
| `static java.lang.String` | `ADD_TO_FILTERED_ROWS` |
| `static java.lang.String` | `ADD_TO_REMOVED_ROWS` |
| `static java.lang.String` | `GENE_ANNOT` |
| `static java.lang.String` | `GENE_ANNOT_FIELD_INDEX` |
| `static java.lang.String` | `GENE_IDENTIFIER_FIELD_INDEX` |
| `static java.lang.String` | `GENE_INFO_FILE` |
| `static java.lang.String` | `GENE_INFO_FILE_SEPARATOR` |
| `static java.lang.String` | `GENE_INFO_HEADERS` |
| `static java.lang.String` | `GENE_INFO_PROBESET_ID_FIELD_INDEX` |
| `static java.lang.String` | `HAS_SNPS_FILENAME` |
| `static java.lang.String` | `HEAT_MAP` |
| `static java.lang.String` | `LOCUS_LINK_FIELD_INDEX` |
| `static java.lang.String` | `MM_POS_FIELD_INDEX` |
| `static double` | `NATURAL_LOG_2` |
| `static java.lang.String` | `NO_LOCUS_LINK_FILENAME` |
| `static java.lang.String` | `NO_SNPS_FILENAME` |
| `static double` | `ONE_MEG` |
| `static java.lang.String` | `ORGANISM` |
| `static java.lang.String` | `PM_MM_RATIO_DATA` |
| `static java.lang.String` | `PROBE_INFO_FILE` |
| `static java.lang.String` | `PROBE_INFO_FILE_SEPARATOR` |
| `static java.lang.String` | `PROBE_INFO_HEADERS` |
| `static java.lang.String` | `PROBE_INFO_PROBESET_ID_FIELD_INDEX` |
| `static java.lang.String` | `PROBE_SEQ_FIELD_INDEX` |
| `static java.lang.String[]` | `separators` |
| `static java.lang.String` | `VIEW_FILTERED_PROBES` |
| `static java.lang.String` | `VIEW_REMOVED_RPOBES` |
| `static java.lang.String` | `WEB_GENE_INFO_FILENAME` |
| `static java.lang.String` | `WEB_SNP_INFO_FILEMANE` |
| `static java.lang.String` | `X_POS_FIELD_INDEX` |
| `static java.lang.String` | `Y_POS_FIELD_INDEX` |


**Constructor Summary**

`Constant()`


**Methods inherited from class java.lang.Object**

`clone, equals, finalize, getClass, hashCode, notify, notifyAll, toString, wait, wait, wait`

**Field Detail**

### separators

```
public static final java.lang.String[] separators
```

---


### GENE\_INFO\_HEADERS

```
public static final java.lang.String GENE_INFO_HEADERS
```

**See Also:**: Constant Field Values

---


### PROBE\_INFO\_HEADERS

```
public static final java.lang.String PROBE_INFO_HEADERS
```

**See Also:**: Constant Field Values

---


### GENE\_INFO\_FILE

```
public static final java.lang.String GENE_INFO_FILE
```

**See Also:**: Constant Field Values

---


### PROBE\_INFO\_FILE

```
public static final java.lang.String PROBE_INFO_FILE
```

**See Also:**: Constant Field Values

---


### GENE\_ANNOT\_FIELD\_INDEX

```
public static final java.lang.String GENE_ANNOT_FIELD_INDEX
```

**See Also:**: Constant Field Values

---


### GENE\_INFO\_FILE\_SEPARATOR

```
public static final java.lang.String GENE_INFO_FILE_SEPARATOR
```

**See Also:**: Constant Field Values

---


### PROBE\_INFO\_FILE\_SEPARATOR

```
public static final java.lang.String PROBE_INFO_FILE_SEPARATOR
```

**See Also:**: Constant Field Values

---


### GENE\_IDENTIFIER\_FIELD\_INDEX

```
public static final java.lang.String GENE_IDENTIFIER_FIELD_INDEX
```

**See Also:**: Constant Field Values

---


### LOCUS\_LINK\_FIELD\_INDEX

```
public static final java.lang.String LOCUS_LINK_FIELD_INDEX
```

**See Also:**: Constant Field Values

---


### GENE\_INFO\_PROBESET\_ID\_FIELD\_INDEX

```
public static final java.lang.String GENE_INFO_PROBESET_ID_FIELD_INDEX
```

**See Also:**: Constant Field Values

---


### PROBE\_INFO\_PROBESET\_ID\_FIELD\_INDEX

```
public static final java.lang.String PROBE_INFO_PROBESET_ID_FIELD_INDEX
```

**See Also:**: Constant Field Values

---


### X\_POS\_FIELD\_INDEX

```
public static final java.lang.String X_POS_FIELD_INDEX
```

**See Also:**: Constant Field Values

---


### Y\_POS\_FIELD\_INDEX

```
public static final java.lang.String Y_POS_FIELD_INDEX
```

**See Also:**: Constant Field Values

---


### MM\_POS\_FIELD\_INDEX

```
public static final java.lang.String MM_POS_FIELD_INDEX
```

**See Also:**: Constant Field Values

---


### PROBE\_SEQ\_FIELD\_INDEX

```
public static final java.lang.String PROBE_SEQ_FIELD_INDEX
```

**See Also:**: Constant Field Values

---


### NO\_LOCUS\_LINK\_FILENAME

```
public static final java.lang.String NO_LOCUS_LINK_FILENAME
```

**See Also:**: Constant Field Values

---


### HAS\_SNPS\_FILENAME

```
public static final java.lang.String HAS_SNPS_FILENAME
```

**See Also:**: Constant Field Values

---


### NO\_SNPS\_FILENAME

```
public static final java.lang.String NO_SNPS_FILENAME
```

**See Also:**: Constant Field Values

---


### WEB\_GENE\_INFO\_FILENAME

```
public static final java.lang.String WEB_GENE_INFO_FILENAME
```

**See Also:**: Constant Field Values

---


### WEB\_SNP\_INFO\_FILEMANE

```
public static final java.lang.String WEB_SNP_INFO_FILEMANE
```

**See Also:**: Constant Field Values

---


### ORGANISM

```
public static final java.lang.String ORGANISM
```

**See Also:**: Constant Field Values

---


### ONE\_MEG

```
public static final double ONE_MEG
```

**See Also:**: Constant Field Values

---


### NATURAL\_LOG\_2

```
public static final double NATURAL_LOG_2
```

---


### HEAT\_MAP

```
public static final java.lang.String HEAT_MAP
```

**See Also:**: Constant Field Values

---


### PM\_MM\_RATIO\_DATA

```
public static final java.lang.String PM_MM_RATIO_DATA
```

**See Also:**: Constant Field Values

---


### GENE\_ANNOT

```
public static final java.lang.String GENE_ANNOT
```

**See Also:**: Constant Field Values

---


### VIEW\_REMOVED\_RPOBES

```
public static final java.lang.String VIEW_REMOVED_RPOBES
```

**See Also:**: Constant Field Values

---


### VIEW\_FILTERED\_PROBES

```
public static final java.lang.String VIEW_FILTERED_PROBES
```

**See Also:**: Constant Field Values

---


### ADD\_TO\_REMOVED\_ROWS

```
public static final java.lang.String ADD_TO_REMOVED_ROWS
```

**See Also:**: Constant Field Values

---


### ADD\_TO\_FILTERED\_ROWS

```
public static final java.lang.String ADD_TO_FILTERED_ROWS
```

**See Also:**: Constant Field Values


**Constructor Detail**

### Constant

```
public Constant()
```


---


|  |  |  |  |
| --- | --- | --- | --- |
| **Overview** \| **Package** \| **Class** \| **Use** \| **Tree** \| **Deprecated** \| **Index** \| **Help** | | |  |
| **PREV CLASS**   NEXT CLASS | **FRAMES**    **NO FRAMES**     **All Classes** |  |  |
| SUMMARY: NESTED \| FIELD \| CONSTR \| METHOD | DETAIL: FIELD \| CONSTR \| METHOD |  |  |


---
